# Two different methods to digitally visualize continuous electrocochleography potentials during cochlear implantation: a first description of feasibility

**DOI:** 10.1007/s00405-023-08400-3

**Published:** 2024-01-03

**Authors:** Theda Eichler, Antonia Lakomek, Laura Waschkies, Moritz Meyer, Nadia Sadok, Stephan Lang, Diana Arweiler-Harbeck

**Affiliations:** Department of Oto-Rhino-Laryngology, Head and Neck Surgery, University Hospital Essen, Essen, Germany

**Keywords:** Intraoperative electrocochleography, Cochlear implantation, Hearing preservation, Digital microscope imaging

## Abstract

**Purpose:**

The study explores the potential of real-time electrocochleographic potentials (ECochG) visualization during electrode insertion using digital microscopes such as RoboticScope (BHS®). Collaborative software development of the MAESTRO Software (MED-EL®) offers continuous ECochG monitoring during implantation and postoperative hearing evaluation, addressing previous time constraints. The study aims to assess software applicability and the impact of real-time visualization on long-term residual hearing preservation.

**Methods:**

Eight patients with residual hearing underwent cochlear implantation with Flex26 or Flex28 electrode according to the Otoplan evaluation. ECochG responses were measured and visualized during electrode insertion, with insertion times recorded. Two randomized display methods (graph and arrows) tracked ECochG potentials. Postoperative behavioral thresholds determined hearing preservation. Successful real-time intraoperative ECochG visualization was achieved in all cases, enabling surgeon adaptation. Mean electrode insertion time was 114 s, with postoperative thresholds comparable to preoperative values. Visualization did not affect surgeon workload. ECochG amplitudes differed between patients with and without residual hearing.

**Conclusion:**

The study demonstrates effective implementation of advanced ECochG software combined with real-time visualization, enabling residual hearing preservation during CI. Visualization had no apparent effect on surgeon performance or workload. Future investigation involving a larger population will assess the long-term impact of ECochG on hearing threshold and structure preservation.

## Introduction

The preservation and protection of cochlear structures during cochlear implantation is fundamental for the preservation of residual hearing. It has been shown that the combination of electrical and acoustic stimulation leads to better speech intelligibility in noise [1, 2], perception of periodicity, sound localization [3] and music perception [4] with cochlear implant (CI). Since 80% of all cochlear implant candidates display low frequency residual hearing [5] the development of methods to preserve cochlear structures has become the focus of scientific attention. Many factors play a role and it is still unclear at what point and for what reason residual hearing disappears. One factor in the reduction of residual hearing is the insertion of the electrode. Due to the opening of the cochlea and a potentially traumatic insertion, approximately 15–20 dB of residual hearing gets lost [6–8]. Therefore, insertion that is as atraumatic as possible is of great interest to all involved. Advances in electrode design such as changes in stiffness, electrode tip, length and diameter, surface morphology, or deployment mechanisms have helped to reduce intraoperative damage to inner ear structures [9, 10]. Adaptation and resulting reduction of electrode length to the length of the cochlea may also lead to improved structural preservation [11, 12].

Monitoring of inner ear function by electrocochleography (ECochG) during electrode insertion appears to be emerging as a sufficient tool for preserving residual hearing and sparing cochlear structures during cochlear implantation [13]. ECochG records electrophysiological responses to acoustic stimulation generated by different components of the inner ear and peripheral cochlear nerve [14]. However, the use of ECochG remains controversial. On the one hand, from a scientific perspective, there exists a certain level of ambiguity regarding the precise interpretation and implications of alterations observed in ECochG measurements [14]. Some studies show that intraoperative loss of amplitude correlates with higher grades of trauma but that such an amplitude loss does not exert any discernible influence on the postoperative hearing threshold [15], while others have shown, that an ECochG amplitude drop of more than 30% leads to a poorer postoperative hearing level [16]. Even if the signal recovers afterwards, it has an impact on the postoperative hearing threshold. One reason for this could be that is a temporary physical contact between electrode and cochlear structures. Another reason might be an destructive interference between hair cells and neural potentials [17]. On the other hand, intraoperative ECochG measurement allows the surgeon to obtain direct feedback on physiological intracochlear activity in case of deterioration, changes in insertion speed, insertion angle or applied pressure [18]. This makes it even more important to track the ECochG potentials adequately and enable a quick reaction to decreasing amplitudes. Feedback can be provided in several ways. Currently, it is common for the audiologist to visually track the ECochG measurement and inform the surgeon in case of deterioration [19]. The audiologist must constantly monitor and interpret the potentials. Since not every potential is remarkable, it is up to the audiological staff to decide which potential changes to pass on to the surgeon. This results in a time delay of multiple seconds. In the meantime, the electrode has been inserted further and the time of loss of intracochlear activity can no longer be directly attributed to a special electrode. Providing direct visual information to the surgeon via the screen, such as it is possible with digital microscopes, avoids the problem of time delay and acoustic degradation and enables the surgeon to react quickly on changes in amplitudes [20]. However, it is necessary to further investigate the usage of ECochG and its impact on postoperative hearing thresholds.

## Material and methods

The goal of this project is, first, to measure ECochG with the new software extension in patients with residual hearing and testing the feasibility of the use of direct visual information to the surgeon via RoboticScope® and picture-in-picture (PIP) mode to transmit ECochG information. Second, ECochG will be measured intraoperatively in all patients at ENT Essen during the project period to obtain norm data on how preoperative residual hearing is related to ECochG measurements and how much residual hearing is necessary to obtain valid measurements. Third, two different types of visualization will be evaluated to obtain information about better feasibility and outcome depending on the visualization method.

*Patients.* Inclusion criteria apart from CI indication were, air conduction thresholds of 80 dB HL or better at least at two low frequencies. In this initial feasibility study, eight subjects (2 f, 6 m) with residual hearing were recruited, including the first surgeries to obtain learning effect. The number of participants will be increased to approximately 50 in the future. Mean age was 57.7 years (SD 9.79 years). The average PTA_low_ (pure tone average of AC thresholds at 125, 250 and 500 Hz) was 68.12 dB HL (SD 15.28 dB HL). Additionally another group of eight patients (mean age 34.6 years, SD 31.1 years) without preoperatively measured residual hearing (mean PTA_low_: 105.2 dB HL) were recruited as a comparative group. All participants gave their informed consent and all tests were performed according to the declaration of Helsinki and were approved by the local ethics committee (reference 20–9695-BO).

*Cochlear implants.* Commercially available Flex Series (MED-EL Medical Electronics, Innsbruck, Austria), Flex28™ and Flex26™, were used according to preoperative Otoplan 3 (CAScination AG, Bern, Switzerland) measurement results and determined hearing loss. Full electrode insertion (minimum 520°) was planned. Intraoperative ECochG was performed in all patients during electrode insertion. Potentials were visualized in real-time via the digital microscope. Patients CT pictures were made with a resolution of 0.16 × 0.16 mm^2^ pixel spacing and slice thickness of 0.6 mm.

*Audiometric tests.* Preoperatively, all patients underwent tone (air (AC))- and bone conduction (BC)) audiometry Additionally, pure tone audiograms tests were performed one day before and one day after surgery (only bone conduction). Intraoperatively, ECochG potentials were detected by using MAESTRO904AS research software (provided by MED-EL®) in addition to regularly performed intraoperative measurements (impedance measurements, electrically evoked stapedial reflex threshold (eSRT) and electrically evoked compound action potential (ECAP)).

**Fig. 1:**
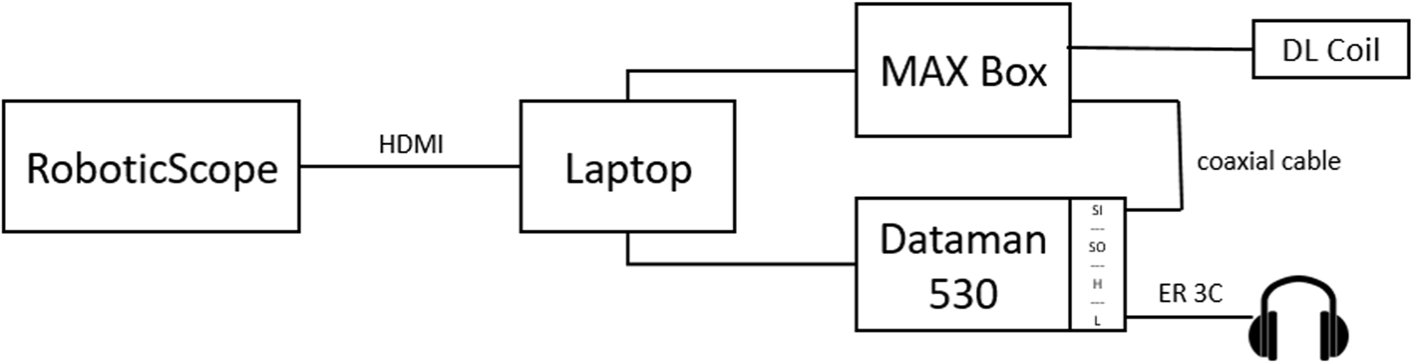
Schematic diagram of the intraoperative setup for the picture-in-picture visualization of the ECochG potentials during CI surgery. The RoboticScope is connected to the laptop via a HDMI cable. The Dataman 530 for the presentation of the acoustic signals over insert earphones and the MAX Box (MED-EL) for the derivation of the ECochG potentials via a DL coil are connected to the laptop. The MAX Box and the Dataman are connected via a coaxial cable

*Intraoperative setting.* The acoustic signals are generated by the DATAMAN-530 Arbitrary Waveform Generator (Dataman Programmers Ltd, Dorset, United Kingdom) and connected to a disposal sound tube (for 3 M E-A-RTONE™ insert earphones, 3 M AEARO) and a disposal eartip (3 M E-A-RLINK™, 3 M AEARO). The insert eartip was positioned in the ear canal before surgery started. Directly preoperatively, the inserted earphone for ECochG measurement was placed under a sterile film drape covering the ipsilateral side of the patient´s face and did not interfere with the surgeon´s field of work. ECochG potentials were derived via the MAX Programming Interface and DL-Coil (MED-EL) and then measured with the MAESTRO904AS research software (Fig. 1).

**Fig. 2:**
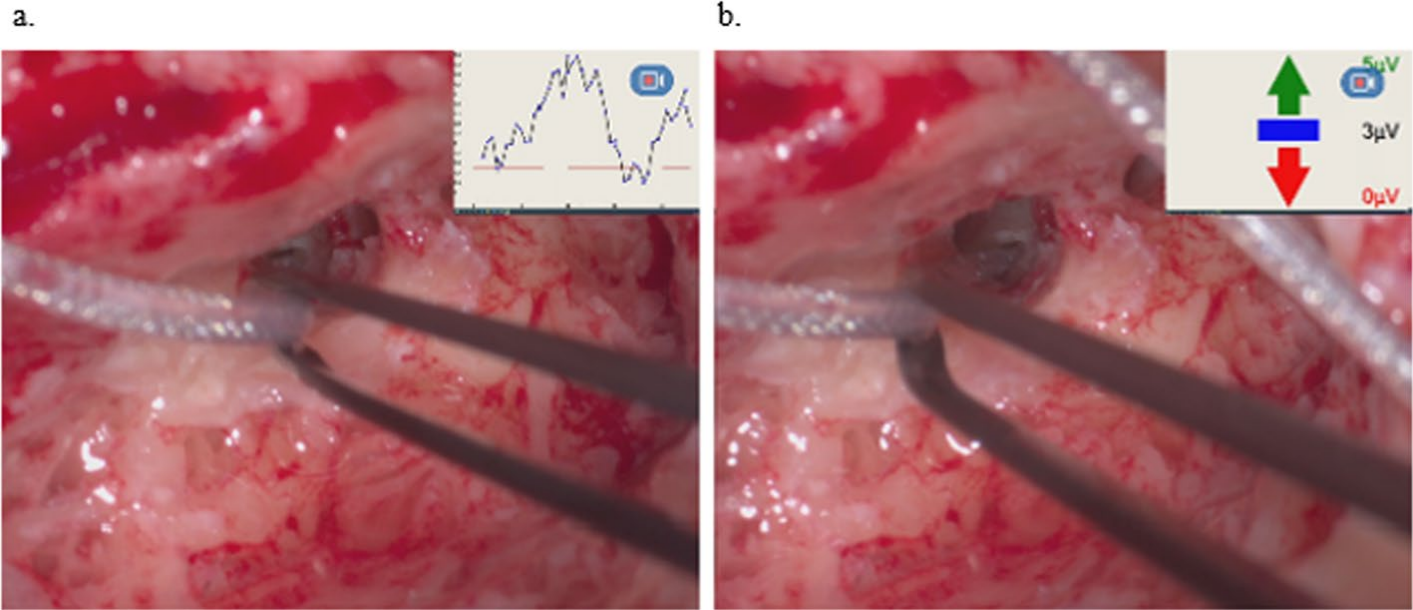
Exemplary screenshots of the surgeons view of ECochG potentials during insertion; **a**: visualization via curve of amplitudes. The system noise level is defined as 1 µV, based on bench data, and displayed as a red dotted line, the potentials are displayed in blue with a black line to connect them; **b**: visualization via arrows. If the potentials are showing an upward trend a green upward pointing arrow is displayed, next to the arrow the maximum amplitude is shown. If the amplitude of the potentials are staying constant a blue bar is displayed, next to the bar the constant value is shown. If the amplitude of the potentials are showing a downward trend a red downward pointing arrow is displayed, next to it the minimum value is displayed. During surgery only one at a time is displayed

Acoustical signals were generated with an 8 ms long tone burst of 250 Hz or 500 Hz and maximum output level of 115 dB, depending on the Otoplan evaluation, with one cycle ramp up and down hamming windowed and two cycles plateau. Default frequency was 500 Hz, but if the AC threshold at 250 Hz, was 20 dB better than the threshold at 500 Hz and the Otoplan evaluation showed that the electrode would affect the area around 500 Hz, the measurements were conducted with 250 Hz. Stimulation rate was variable at about 25 Hz. During insertion of the electrode array, the FFT amplitude of cochlear microphonics corresponding to the first harmonics of the tone burst frequency was continuously measured. Each ECochG signal was the average of 100 sweeps, band-pass filtered from 50 Hz to 10 kHz. For recording, the most apical electrode E1 was used, while the ground plate on the implant housing serve as a reference and ground electrode. A second screen was generated and transfered to the digital microscope RoboticScope® (BHS Technologies GmbH, Innsbruck, Austria). The potentials could be visualized as picture-in-picture in one corner of the screen enabling the surgeon to receive direct visual feedback during electrode insertion (Fig. 2).

To depict the recorded ECochG potentials, two different views are available. On the one hand, a curve of the amplitudes of the ECochG potentials at each measuring time can be displayed, on the other hand arrows (up, down, constant) can be presented, which only visualize the trend of the last 10 averaged time points. Warning message is displayed in case CI coil is accidentally removed from the implant and recording is automatically stopped. Additionally, an objective audiogram was determined at the end of insertion. Right after surgery the surgeon had to complete the NASA Task Load Index (TLX) questionnaire to evaluate the visualization in terms of workload. NASA TLX is a subjective workload assessment tool which allows users to perform subjective workload asessments on operator(s) working with various human–machine interface systems. It captures workload at multiple levels: mental demands, physical demands, time demands, performance, effort and frustration [21, 22]. Two experienced surgeons were included in the study to ensure comparability between the surgeries and to enable the observation of learning effects. The different visualization methods were presented randomized between the surgeons.

*Data analysis.* Graphs and statistical analysis were performed using Microsoft Excel 2016 (Microsoft Corporation, Redmond, WA, USA) and MATLAB 2022a (The MathWorks, Inc., MA, Natick, USA). For statistical analysis the non-parametric Mann–Whitney-U test was used.

## Results

**Fig. 3:**
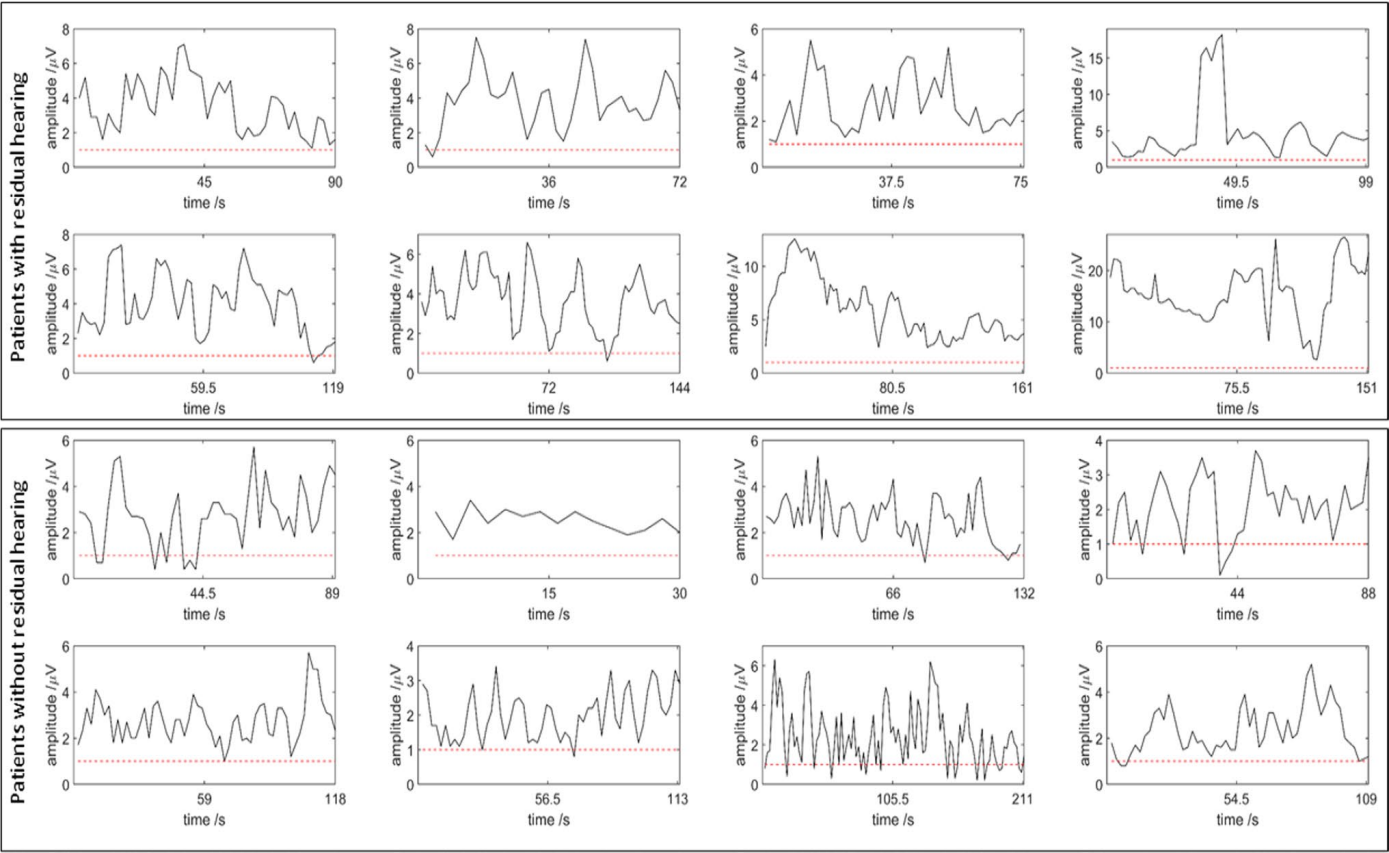
Comparison of ECochG potentials of patients with (upper box) and without (lower box) residual hearing. The *y*-axis shows the FFT amplitude of the first harmonic in µV and the *x*-axis the time in seconds. System noise is the horizontal dashed red line. The higher the amplitude, the higher the amount of measured ECochG potentials

*Feasibility of the intraoperative setting.* The RoboticScope® is controlled by head movement via a head-mounted display. This means that the surgeon is not forced to take his hands out of the operating field to change settings (zoom, section etc.). Due to the completely different handling compared to other (digital) microscopes, there were operating difficulties, especially with the first applications. However, the handling improves with each application, so that the full potential can be exploited. ECochG potentials were visualized in the upper right corner of the surgeons screen, but are not visible for anyone else to date. Four surgeries were performed using the graph visualisation, leading to a slightly higher, but not significant (*z* = 0.146, *p* = 0.88), mean workload (*N* = 4, unweighted NASA-TLX mean score: 31.8, SD 16.8) than the four surgeries using the arrow visualisation (*N* = 4, unweighted NASA-TLX mean score: 28.3, SD 22.6). Both mean values indicate a medium workload [23], therefore the evaluation shows no trend regarding an increased workload with either of the two visualization methods so far. However, to evaluate the ECochG potentials as a tool to observe residual hearing, ECochG potentials were also measured in a small group without preoperatively measured residual hearing. The resulting ECochG potentials of both groups are shown in Fig. 3. In patients without residual hearing, the amplitude of the potentials fluctuate around the noise level (mean: 2.47 µV) or show strongly varying amplitudes, while in patients with residual hearing, considerable higher amplitudes of potentials (mean: 6.08 µV) are recorded. The difference between the two groups is highly significant (*z* = 15.22, *p* < 0.001). The ECochG amplitudes during insertion can be categorized according to [24]. Type A represents an overall increase in amplitude from the beginning of insertion to completion, while the amplitudes of Type B have their maximum close to the beginning and decrease during insertion until completion with an occasional complete loss of a signal. Type C is described as similar amplitudes at the beginning and at the end of the insertion, with a maximum amplitude reach mid-insertion [24]. The categorization of the first patients is displayed in Table 1.

**Table 1:** Clinical (Age, Sex) and audiometric characteristics of the included patients. The Otoplan 3 measured cochlear diameter in mm and the according chosen electrode. Air conduction (AC) thresholds one day before surgery at 125, 250, 500 and 1000 Hz. Bone conduction (BC) thresholds in dB HL one day before and one day after surgery at 250, 500 and 1000 Hz. BC thresholds out of audiometer limits are marked with a dash. Insertion time in seconds from first electrode contact to full insertion and categorization according to Harris et al. (2021)

Age	Sex	Cochlear Diameter	Electrode	AC pre 125 Hz	AC pre 250 Hz	AC pre 500 Hz	AC pre 1000 Hz	BC pre 250 Hz	BC pre 500 Hz	BC pre 1000 Hz	BC post 250 Hz	BC post 500 Hz	BC post 1000 Hz	Insertiontime [s]	Harris Type [24]
50	M	9.62	Flex28	70	75	75	70	–	–	65	–	–	65	90	C
59	M	9.24	Flex28	70	85	75	100	–	–	–	–	–	–	72	C
69	M	10.63	Flex26	75	85	75	95	–	–	–	–	–	–	75	C
62	M	9.22	Flex28	75	70	75	70	40	55	60	35	65	70	100	C
49	M	9.29	Flex28	75	75	75	75	45	–	70	45	–	70	119	C
74	M	9.3	Flex28	40	45	75	80	35	60	75	40	60	75	144	B
56	F	9.99	Flex28	30	35	75	85	30	–	75	25	–	80	161	B
43	F	9.51	Flex28	40	55	75	75	40	60	70	40	60	–	151	A

*Insertion time.* Insertion time was defined as the time between first cochlear electrode contact and end of insertion, stated by the surgeon. Mean insertion time was 114 s (SD 32.7 s) over all visualizations. When using the visualization method graph the mean insertion time was 110 s (*N* = 4; SD 28.74 s), using arrows the mean insertion time extends to 117.5 s (*N* = 4; SD 35.9 s). Due to continuous visual feedback, independent of the visualization, the insertion process was more even and controlled and therefore slower than during implantation in conventional technique (average 30–50 s [9]). Visualizing the ECochG potentials via arrows led to a slightly longer, but not significant (*z* = – 0.144, *p* = 0.88), insertion time compared to the graph.

*Hearing preservation.* Hearing preservation was first evaluated via bone conduction threshold one day after surgery. The average preoperative PTA_bc_ (pure tone average of bone conduction at 250, 500 and 1000 Hz) over all patients with a measurable BC threshold (*N* = 6) was 55.71 dB HL (SD 14.62 dB HL). One day postoperatively the PTA_bc_ lowered to 56.15 dB HL (SD 16.54 dB HL). Table 1 displays the individual levels of hearing one day prior and one day after the surgery. Hearing preservation was possible in all measured patients. Except for one patient, hearing thresholds of bone conduction did not decrease more than 10 dB across all frequencies. In the calculation of the average, missing values were systematically disregarded, potentially introducing bias and compromising the accuracy of the derived measure due to an incomplete representation of the dataset. These are preliminary results at an early stage after surgery. Residual hearing will be re-evaluated six weeks, 4 and 7 months after surgery with an additional audiometric testing and ECochG measurement.

## Discussion

The present study aims to evaluate the software extension from simple long-lasting conduction of auditory brainstem response measurement for each electrode to a continuous ECochG measurement [19] during insertion in combination with a real-time visualization in a digital microscope and PIP mode regarding its feasibility. In this study, the RoboticScope was used as a digital microscope to apply PiP technology. This is also possible with other digital microscopes such as the Arriscope and leads to equivalently good outcomes with regard to the preservation of residual hearing [20]. The PiP application in the RoboticScope was newly implemented within the scope of the study and was therefore part of the feasibility study. Especially the future use of robotic surgery during cochlear implantation will require reliable and objective measurements to maintain the quality standard [25]. Accordingly, research is being conducted on possibilities to objectively monitor the insertion. In addition to the ECochG, there is for example the SmartNav tool (Cochlear Ltd., Sydney, Australia). SmartNav allows the measurement of the insertion speed as well as the determination of the insertion angle [26]. The insertion angle sensitivity is indicated with ± 45° and insertion measurements can only be performed with straight electrodes [27]. The measurement is so far only visible to the audiologist and due to the division of the screen unsuitable for the PiP technology. Accordingly, the tool is not yet sufficiently developed to be considered as a reliable tool for monitoring electrode insertion. Therefore, it is important to evaluate whether and how the presented ECochG measurements work.

A second objective of the study, which goes hand in hand with the first objective, is to investigate the influence of controlled insertion via PiP visualization on the preservation of residual hearing and cochlear structures. Even though shorter electrodes can also be used on their own to preserve low-frequency residual hearing, ECochG remains a useful tool for monitoring insertion. Along with PiP mode of digital microscopes, the surgeon is able to insert more gently and react quicker to changes of ECochG potentials [28]. Combining shorter electrodes and ECochG may be a good solution to provide satisfying outcomes. Prior scientific investigations have provided evidence indicating that distinct patterns of ECochG responses are reflected in the outcome of hearing thresholds [24, 29]. The Harris pattern Type A, an overall increase in amplitude from the beginning of insertion until completion, seem to be the most common in literature for patients with good residual hearing, whilst patients with a poorer residual hearing (but not totally deaf) tend to show a pattern of Type C, maximum amplitude reached mid insertion [24]. However only a small patient population was considered, which makes it even more important to evaluate if these patterns are reproducible in a larger collective and which impact they have on postoperative hearing thresholds. So far the Type C pattern has been measured in the majority of patients, which is in contrast to the literature [24]. However, this observation may be attributed to a potentially broader inclusion criterion in regards of preoperative hearing thresholds and needs to be further investigated. Nevertheless, it could be shown that the experimental setup, including the extension of the ECochG measurement, operates successfully and seems to be a sufficient tool to provide a real time feedback to the surgeon during electrode insertion. However, it is evident that the better the preoperative AC threshold the better the measured ECochG potentials can be interpreted. To evaluate a hard criterion on how much residual hearing is necessary to obtain valid ECochG measurements, more data needs to be collected.

In prospective applications, ECochG holds promise as a prospective tool capable of not only monitoring residual auditory function but also facilitating the tracking of intra-cochlear traumas, such as scalar translocations or electrode misinsertions (e.g., tip fold-overs). Currently, only a limited number of studies have explored the association between ECochG amplitudes and scalar electrode positions. Enhanced specificity and sensitivity in the accurate identification of scalar translocations necessitate the integration of phase characteristics with amplitude measurements. A phase inversion could be due to biomedical changes of the basilar membrane [30], which could possibly also be used as an indicator for folded over electrodes [31]. Notably, postoperative alterations in cochlear physiology, occurring in the interval between electrode implantation and activation, are more conspicuous in the case of electrodes that translocate into the scalar vestibuli [18]. Consequently, it is imperative to advance ECochG measurement techniques and visualizations to enhance the detection of scalar alterations.

Even though there are amplitude drops observed in the ECochG thresholds, the use of PiP with a digital microscope results in satisfying hearing outcomes. Residual hearing in bone conduction could be preserved in almost all patients which goes along with the previous research on that field, which have been shown significant correlations between intraoperative and postoperative hearing thresholds [20, 32]. This contradicts the findings of [16], who had shown that drops in amplitude correlate with a poorer hearing threshold. Additional behavioral data have to be obtained and compared to postoperative ECochG thresholds, since there is only limited amount of data at the moment. However, the presentation of ECochG potentials in the tested software was successfully achieved and has been shown to be an indicator of the patient's residual hearing. The difference in amplitudes between patients with and without residual hearing can clearly be seen.

Third, two different visualization methods were used to present the ECochG potentials to the surgeon, to evaluate if the workload is higher with one of the two visualization methods or if they can be seen as equivalent and if its influences the postoperative hearing threshold. The data of the NASA-TLX workload questionnaire indicates that the workload is similar for both visualization methods independent of the surgeon. Nevertheless, the insertion time is a little longer when using the arrows, which could imply an increased level of attention, although it was expected the other way round, since the arrows only show an already analyzed version of the ECochG potentials, while the graph needs to be further cognitively interpreted. First results show no influence of the visualization method on the hearing outcome. Collecting more data will show if this trend continues and if there is a correlation between visualization method and preservation of residual hearing.

To our knowledge, this is the first study describing the usage of the RoboticScope during cochlear implantation using the picture in picture mode for ECochG guided electrode insertion. These are the initial findings from a small patient cohort using digital real-time ECochG potential imaging during surgery. As of right now, it is difficult to estimate the long-term implications of changing insertion way and speed on hearing preservation. However, these data demonstrate that intraoperative ECochG in the surgeon's field of view performed with real-time digital visualization can facilitate real-time feedback during the insertion period. To evaluate long-term impacts on residual hearing and consequences on patients' outcomes, additional research will be conducted.

## Data Availability

Not applicable.
